# Human Amniotic Fluid Cells Form Functional Gap Junctions with Cortical Cells

**DOI:** 10.1155/2012/607161

**Published:** 2012-06-26

**Authors:** Anna Jezierski, Kerry Rennie, Roger Tremblay, Bogdan Zurakowski, Andreé Gruslin, Marianna Sikorska, Mahmud Bani-Yaghoub

**Affiliations:** ^1^Neurogenesis and Brain Repair Group, Neurobiology Program, Institute for Biological Sciences, National Research Council Canada, Ottawa, ON, Canada K1A 0R6; ^2^Department of Cellular and Molecular Medicine, Faculty of Medicine, University of Ottawa, Ottawa, ON, Canada K1H 8M5; ^3^Department of Obstetrics and Gynecology, Faculty of Medicine, University of Ottawa, Ottawa, ON, Canada K1H 8M5

## Abstract

The usage of stem cells is a promising strategy for the repair of damaged tissue in the injured brain. Recently, amniotic fluid (AF) cells have received a lot of attention as an alternative source of stem cells for cell-based therapies. However, the success of this approach relies significantly on proper interactions between graft and host tissue. In particular, the reestablishment of functional brain networks requires formation of gap junctions, as a key step to provide sufficient intercellular communication. In this study, we show that AF cells express high levels of CX43 (GJA1) and are able to establish functional gap junctions with cortical cultures. Furthermore, we report an induction of Cx43 expression in astrocytes following injury to the mouse motor cortex and demonstrate for the first time CX43 expression at the interface between implanted AF cells and host brain cells. These findings suggest that CX43-mediated intercellular communication between AF cells and cortical astrocytes may contribute to the reconstruction of damaged tissue by mediating modulatory, homeostatic, and protective factors in the injured brain and hence warrants further investigation.

## 1. Introduction

Recent advances in regenerative medicine have boosted efforts to explore the therapeutic potentials of stem cells to repair damaged tissue in the injured brain (reviewed in [1–4]). In particular, the transplantation of embryonic stem cells [5], fetal neural stem or progenitor cells [6–8], or bone-marrow-derived stem cells [9, 10] into the injured brain has been explored extensively. However, human embryonic stem (ES) cells and fetal neural stem cells are subject to ethical considerations and the risk of tumor development, whereas adult neural stem cells have limited proliferation capabilities and lineage restriction. Therefore, other stem cell sources, such as human amniotic fluid (AF) [11–13], are being considered for therapeutic applications. There is evidence that AF contains stem cell subpopulation(s) [14] isolated based on c-Kit (CD117–the receptor for stem cell factor [15]) expression, which share some of the characteristics of embryonic and adult stem cells [14]. For instance, several reports have shown that AF cells can differentiate along the adipogenic and osteogenic [16–18], myogenic [19, 20], and endothelial [21] pathways. Furthermore, AF cells have also been shown to harbour the potential for neurogenic differentiation, using different induction protocols [14, 18, 22–25]; however, the proof that these cells can differentiate into functional neurons remains elusive [26, 27].

Nonetheless, the versatility of AF-derived cells for therapeutic applications has been investigated in various animal injury models in the central and peripheral nervous system [14, 28–32]. Although it has been suggested that AF-derived cells exert beneficial effects on the ischemic brain to an extent comparable with the neuroprotective effect of embryonic neural progenitor cells [32], it remains to be determined whether or not these cells are capable of integrating into the brain and developing functional connectivity with the host tissue to support neuroregenerative and protective capabilities. The success of this strategy depends on the formation of a rapid and efficient intercellular communication between grafted AF cells and the host tissue followed by the reestablishment of functional networks. In fact, a recent report by Jäderstad et al. [33] clearly shows that an essential step in the functional integration of grafted ES cells, even before mature electrochemical synaptic communication, is cell-cell coupling via gap junctions. This integration is, at least in part, dependent on the formation of gap junctional intercellular communication (GJIC), which is considered to be an indispensable mechanism for the propagation of information among cells in the CNS. Gap junctions are composed of two juxtaposed, membrane-bound connexin hemichannels; each composed of six connexin subunits, which are joined to bridge the cytoplasm of two neighbouring cells [34, 35]. This consolidation allows the transfer of small ions and molecules, nutrients, metabolites, second messengers, and more recently miRNAs [34, 36]. Hence, intercellular communication between graft and host cells underlies many of the early cellular interactions and plays a central role in the rescue of damaged host cells after brain injury [33]. It is expected that intercellular gap junction formation would result in cell-cell communication between host and graft cells and hence increase transplantation success rates as well as the transfer of therapeutic agents. More specifically, connexin-associated gap junction formation and function have been shown to be pivotal for ensuring host cell well-being and potentially mediating a neuroprotective effect [33]. In fact, NSC-mediated rescue of damaged host neurons did not occur when gap junction formation was suppressed by pharmacological and/or RNA-inhibition strategies [33].

Although AF cells have been previously transplanted into several tissues, including the brain, currently there is no information on gap junctions in these cells and whether they form a means of intercellular communication with the host tissue. Therefore, a better understanding of the interactive processes by which AF cells integrate into host neural tissue may provide insights into the interplay between donor and recipient. In this study, we examine the expression of connexins in AF cells at the RNA and protein levels, using *in vitro* and *in vivo* techniques. In addition, we determine whether AF cells can form functional gap junctions with other AF cells as well as with cortical cells.

## 2. Methods

### 2.1. Cell Culture

Human amniotic fluid (AF) cells were obtained from the Ottawa Hospital, General campus (Ottawa, ON, Canada), following amniocentesis in women at 15 to 35 weeks of gestation (AF15–AF35). The study was approved by the Ottawa Hospital and the National Council Canada-Research Ethics Boards, and a written informed consent was obtained from each donor. AF cells were cultured in Dulbecco's modified Eagle medium (DMEM, Invitrogen) supplemented with 20% fetal bovine serum (FBS, Hyclone) and maintained at 37°C and 5% CO_2_ (as described in [37]). AF cells were passaged at 70% confluency every 2-3 days, using 0.05% trypsin/EDTA (Invitrogen) at a 1 : 3 split ratio.

To generate AF-derived single cell clones [37], a single cell suspension was prepared by gentle trypsinization, and individual AF cells were deposited one cell per well of a 96-well plate, containing 100 uL of DMEM + 20% FBS, using a MoFlo cell sorter (Beckman Coulter). Once the cultures became 70% confluent, clones were subcultured first into 24-well plates, followed by 6-well plates (Nunc), and eventually into 10 cm culture plates (Corning), using the above-mentioned conditions. To purify c-kit-positive AF cells from AF cultures, dissociated single AF cells were stained with c-kit antibody (Santa Cruz, sc-5535) for 30 minutes at 4°C, as previously described [14]. Following the incubation period, the cells were washed twice with cold 2% FBS in PBS and incubated for 30 minutes at 4°C with a secondary phycoerythrin- (PE-) conjugated antibody. The cells were subsequently washed, resuspended in 2 mL of cold 2% FBS in PBS, filtered through a 70 *μ*m filter, and analyzed using a MoFlo Cell Sorter (Beckman Coulter). The clonal (AF-F5) and c-kit-positive AF cells were thereafter expanded serially with a split ratio of 1 : 3 and cultured in DMEM containing 20% FBS to establish the AF-F5 single-cell-derived clonal line and c-kit-positive AF cell population.

Mouse cortical progenitors were isolated from the E13 ventricular zone, plated onto PLL-coated coverslips (9 × 10^5^ living cells/mL) in DMEM + 10% FBS, and examined within 24 hrs after plating, as previously described [38]. Cortical neurons were generated from neural progenitors by reducing the serum concentration (i.e., 0.5% FBS) during the first 24 hrs, followed by treatment with DMEM + N2 supplement to limit the generation of glial cells. Medium was replenished every 48 hrs for 7 days. Astroglial cultures were generated from E13 neural progenitors (0.5–5 × 10^5^ living cells/mL) cultured in DMEM + 10% FBS. Medium was replenished every 48 hrs for 3 weeks, and cells were passaged several times to eliminate neurons in the cultures [38]. To generate mixed cultures of cortical neurons and astrocytes, E13 neural progenitors were maintained in DMEM + 10% FBS for 2 weeks without passaging.

NT2-D1 progenitor cells (ATCC) were cultured in DMEM (Invitrogen) media supplemented with 10% FBS (Hyclone). Pure cultures of NT2-derived neurons (NT2-N) were prepared as previously described [39]. HaCaT cells were a generous gift from Dr. Kursad Turksen (Sprott Centre for Stem Cell Research, Ottawa, ON, Canada) and were cultured in DMEM (Invitrogen) supplemented with 10% FBS (Hyclone) and split every 2 days.

### 2.2. RNA Extraction and RT-PCR

Total RNA was extracted from cells, using TriReagent (Molecular Research Centre), as previously described [37]. Total RNA was quantified with NanoDrop (Thermo Fisher Scientific), and 1 *μ*g was reverse transcribed using Quantitect reverse transcriptase (Qiagen). RT-PCR amplifications were carried out using iQ Supermix (Bio-Rad) in a 20 *μ*L volume containing, 5 *μ*M sense and antisense primers (Table 1), and 15 ng of cDNA. The PCR program consisted of a denaturing step for 3 mins at 94°C, followed by 30 secs at 94°C, 30 secs at 55–58°C, and 30 secs at 72°C for 40 cycles. The final PCR extension period was 5 mins at 72°C. PCR products and 1kb ladder (Invitrogen) were separated on a 2% ethidium bromide agarose gel, and the images were captured with FluorChem 8900 Imager (Alpha Innotech). The amplicon size was confirmed by comparison with the ladder (Invitrogen). Expected amplicon sizes are shown in Table 1. *B-ACTIN *(*ACTB*) was used as a normalizing gene. NT2/D1, HaCaT, and NT2-neurons (NT2-N) were used as positive controls.

### 2.3. Antibodies

The following antibodies were used in this study: *β*-ACTIN (1 : 5000, WB, Sigma), Cx43 (1 : 500, ICC; WB, 1 : 4000, ICC, Sigma), Cx26 (1 : 100, Zymed), GFAP (1 : 200, ICC, NeoMarkers), GFAP (1 : 200, ICC, DAKO), Golgin-97 (1 : 200, ICC, Molecular Probes), MAP2 (1 : 200, ICC, Sigma), human nuclear antigen (1 : 100, ICC, antibodies-online), human mitochondrial marker (MTCO2) (1 : 50, ICC, Abcam), c-kit antibody (1 : 100, FACS, Santa Cruz sc-5535), and fluorescence-conjugated secondary antibodies (Alexa Fluor 488 anti-rabbit or mouse, rhodamine anti-mouse and Alexa 647 anti-mouse, 1 : 500, molecular Probes). Hoechst (1 : 1000, Sigma) was used to stain nuclei.

### 2.4. Western Blotting

Cells were washed with cold TBS and lysed directly in the culture plate using ice-cold lysis buffer (25 mM Tris-HCL, pH 7.6, 150 mM NaCl, 1% Triton-X, 1% Na deoxycholate), containing a protease inhibitor cocktail (Roche). Cell lysates were incubated for 30 min on ice and clarified by centrifugation at 20 000 ×g at 4°C for 20 mins. Protein samples (40 ug) and a molecular weight rainbow marker (Amersham) were electrophoresed on a 10% sodium dodecyl sulfate-polyacrylamide gel (SDS-PAGE) and transferred to a nitrocellulose membrane (Amersham), using a wet transfer apparatus (Bio-Rad) at 20 V overnight at 4°C. The membranes were incubated in TBS containing 5% nonfat milk with 0.1% Tween-20 (Sigma) for 1 hr at room temperature to block nonspecific binding and then incubated in primary antibodies overnight at 4°C. The membranes were then washed three times for 10 mins with TBS containing 0.1% Tween-20 and incubated with a peroxidase-conjugated secondary antibody for 1 hr at room temperature. Immunoreactivity was visualized, using chemiluminescent substrate (New England Nuclear) and captured by FluorChem 8900 (Alpha Innotech).

### 2.5. Immunocytochemistry

Cells were grown on coverslips, washed with PBS, and fixed with 65% ethanol containing 0.15 M NaCl for 20 mins [37]. For staining with human nuclear antigen, cells were fixed with 3% paraformaldehyde for 5 mins, washed twice with PBS, and permeabilized for 20 mins in 0.2% Triton-X in PBS (pH 7.0). Following fixation, the coverslips were blocked with serum-free-protein block (Dako) for 30 mins and incubated for 1 hr at room temperature with the primary antibody. Following three subsequent washes (5 mins each) in PBS, the coverslips were incubated with a fluorescence-conjugated secondary antibody for 1 hr, washed, and counter-stained with Hoechst (Sigma). The coverslips were mounted, using Vectashield mounting medium (Vector Laboratories), and immunoreactivity was examined under an Axiovert 200 M fluorescence microscope (Zeiss) and a confocal microscope (Olympus).

For GFAP, Cx43, and Hoechst staining in sections containing implanted AF-DsRed cells, Alexa 488 and Alexa 647 were used as the secondary antibodies to visualize Cx43 and GFAP, respectively. In addition, different filter sets were used for Hoechst (excitation 365, emission 420) and AF-DsRed, cells (excitation 546, emission 575). A separate image was acquired for each fluorophore (Hoechst, Alexa 488, DsRed and Alexa 647), using a laser scanning confocal microscope (Olympus FluoView with BX61 microscope), and the four images were superimposed, using Adobe Photoshop.

In the sequential quadruple staining of AF cells with Cx43, GFAP, human nuclear antigen (hNuc), and Hoechst, cells were first stained with Cx43 and hNuc antibody, washed, and preincubated with a mouse Ig blocking reagent (Vector Laboratories) to reduce undesired binding of the subsequent antibody staining for GFAP detection. The cells were then counterstained with Hoechst.

### 2.6. Dye Coupling

Dye coupling experiments were performed to evaluate the functionality of gap junctions between individual AF cells and other AF cells in culture or mouse neural progenitors, neurons, and astrocytes, as previously described [39, 42]. In brief, AF cells were preloaded with two dyes: 0.1% 1-1′-dioctadecyl-3,3,3,3-tetramethylindocarbocyanine percholate (DiI, Invitrogen) and 0.1% calcein-acetoxymethyl ester (Calcein AM, Invitrogen) for 20 mins at 37°C. DiI, a lipophilic dye that binds to cell membranes and is not transferred to adjacent cells, was used to label donor cells. Calcein AM is in a membrane-permeant form and is taken up by donor cells and hydrolyzed to calcein (MW = 623) by cellular esterases. After cleavage, calcein was readily transferred to adjacent receiving cells through gap junction channels. The DiI- and calcein-loaded cells were washed with isotonic glucose solution to remove excessive dye and dissociated into a single cell suspension, following incubation in trypsin-EDTA (Invitrogen) for 2 mins. Preloaded single AF cells were plated onto cultures of AF or mouse cortical neural progenitors, neurons, or astrocytes, and dye coupling was evaluated after 4 hrs. The level of coupling was determined by counting calcein-positive, DiI-negative cells coupled to a calcein-positive, DiI-positive cell, and the data was presented as mean ± SEM.

### 2.7. Scratch Wound Injury

Scratch wound procedure was performed as previously described [43]. Briefly, scratch wound injury was applied to confluent cortical cultures grown on coverslips, using a 21^1/2 ^G needle (0.8 mm diameter) to create a wound. The cells were washed three times with culture media, fixed with 65% ETOH + 0.15 M NaCl without injury, or 15 mins, 24 hrs, 48 hrs, or 72 hrs after injury. In some experiments, AF cells were plated onto cortical cultures immediately following injury, and the cocultures were fixed at the above-mentioned time points. The labeling of AF cells with EGFP was performed, as previously described [37].

### 2.8. Motor Cortex Brain Injury

Brain injury studies were approved by the Animal Care Committee at the National Research Council Canada. Briefly, 6-week-old C57 black mice (C57Bl/6, Charles River) weighing about 25 g were anesthetized with isoflurane (Aerrane, Baxter) and placed into equal groups: injury or no injury with cell injection or implant. Prior to injury, mice were placed in a stereotaxic frame, and a midline incision was made in the skin to expose the skull. The bone overlying the motor cortex was removed with a dental drill following mapping, using specific stereotaxic coordinates (from “AP −0.25 mm to −1.0 mm, Lat +0.7 mm”, to “AP +1.25 mm to +3.0 mm, Lat +2.4 mm”) with respect to Bregma (0 mm), as previously described [44]. Injury to the left motor cortex was performed using a sterile graduated needle to remove neural tissue to a depth of 1 mm. The injury site was sealed with bone wax, covered with topical anaesthetic (0.50% marcaine bupivacaine hydrochloride, Sigma), and the skin was sutured.

For transplantation studies, AF cells engineered to express DsRed driven from a CMV promoter by lentiviral infection (Tet07-CMV-DsRed) were injected into three sites within the motor cortex (100,000 cells in 2 *μ*L of PBS per injection), using a 10 *μ*L Hamilton syringe, controlled by an infusion pump at a constant speed (0.5 *μ*L/min) over 4 mins. The syringe was held in place for 5 mins and then gradually withdrawn. After the procedure, bone wax was used to seal the injection sites and the skin was sutured.

The animals were allowed to recover in their cages and sacrificed 2 weeks later. The brains were removed and processed, as described below. The animals were sacrificed after 12 days and the brains were perfused, removed and fixed with 4% paraformaldehyde in PB overnight, washed twice with PB and transferred to 30% sucrose in PB for 2 days. The brains were frozen in O.C.T. compound and sectioned into 8 *μ*m slices (Leica CM 1950). Prior to staining, the sections were thawed at room temperature for 15 mins, washed three times (5 min each) in PBS, and immunostained, as described earlier. Injected AF cells were identified based on DsRed expression (AF-DsRed).

## 3. Results

### 3.1. AF Cells Predominantly Express Connexin 43 (CX43)

**Figure 1:**
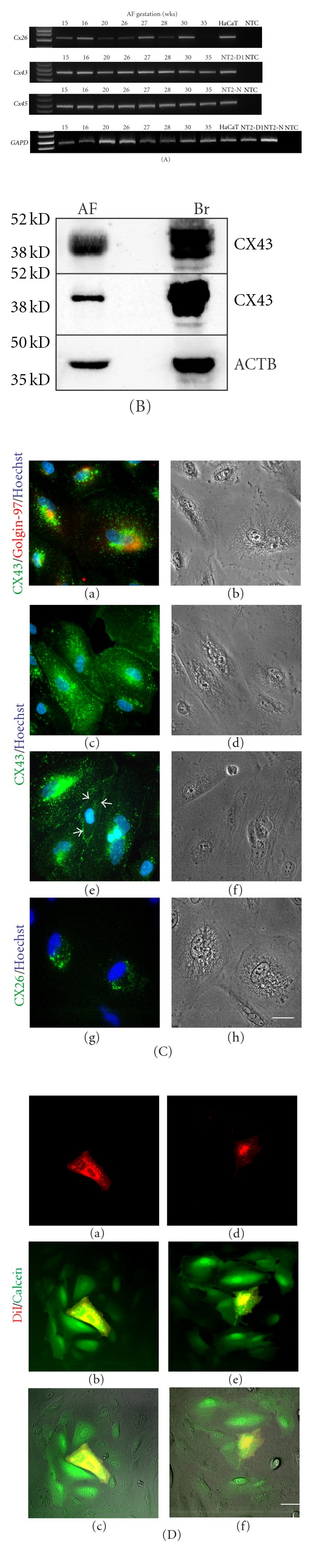
Expression of connexins in human amniotic fluid (AF) cells. Figure 1(A): RT-PCR analysis of Connexin (*CX*) expression in AF cells at 15 to 35 weeks (wks) gestation. AF cells expressed *CX26*, *CX43*, *CX45 *in all gestation periods examined. *GAPDH* transcript and human HaCaT, NT2-D1, and NT2-N cells were used as internal and positive controls, respectively. NTC, No Template Control. HaCaT, Human keratinocyte cell line; NT2-D1, (NTera-2) human teratocarcinoma cell line and NT2-derived neurons (NT2-N). Figure 1(B): Western blot analyses confirmed the expression of CX43 protein in AF26 (top panel) and AF30 (middle panel) cells. Embryonic day 18 (E18) mouse brain (Br) and B-ACTIN (ACTB) were used as positive and internal controls, respectively. Figure 1(C) Immunocytochemistry further verified the presence of CX43 and CX26 proteins in AF cultures. CX43 expression (green) was detected as punctate staining at the perinuclear region and the cell membrane. CX43 appeared to be associated with golgi complex in the perinuclear region, as determined by golgin-97 (red) and CX43 double staining (a). The punctate staining pattern ((a), (c), (e)) demonstrated the dynamic translocation of CX43 protein from Golgi complex (a) to the cell membrane ((e), arrowheads). Unlike CX43, CX26 protein expression appeared limited to the perinuclear region (g). Nuclei were stained with Hoechst (blue). Panels (b), (d), (f) and (h) represent the corresponding phase contrast images of (a, c, e and g); respectively. Scale bar: 25 *μ*m. Figure 1(D) Dye coupling assessment in AF cells. AF donor cells (AF26, (a); AF16, (d)) were preloaded with DiI (red) and calcein (green) and plated as single cell suspensions onto confluent monolayers of receiving AF cells. Calcein transferred from the donor AF cell to adjacent receiving cells, indicating the formation of functional gap junctions between individual AF cells within 4 hours. Scale bar: 50 *μ*m.

To establish a profile of connexin expression in AF cells, we performed RT-PCR to examine the expression of connexins commonly expressed in the brain (*CX26, CX30, CX32, CX36, CX37, CX40, CX43*, and *CX45)*. Our results show that AF cells ubiquitously expressed *CX43* (*GJA1*) and *CX45* (*GJA7*) at the gestation periods examined (AF15–AF35) (Figure 1(A)). The expression of *CX30*, *CX32*, *CX36*, *CX37*, or *CX40* was not detected in any of the gestation periods examined (data not shown), whereas *CX26* (*GJB2*) RNA was expressed in the majority of gestation periods (Figure 1(A)) and CX26 protein was only found in a small subset of AF cells in culture (Figure 1(C), (g)-(h)). Of the connexins expressed, CX43 was the most abundant protein in AF cells, as determined by western blotting and immunocytochemistry (Figure 1(B) and 1(C), (a)-(f)). Similar to other connexins, CX43 is assembled into connexins in the *trans*-Golgi network and transported to the cell membrane where adjacent hemichannels on apposed cells dock to form gap junction plaques [45]. Indeed, we found an intracellular pool of CX43 in the perinuclear Golgi apparatus, as confirmed by *trans*-Golgi network membrane protein golgin-97-positive staining in AF cells (Figure 1(C), (a)-(b)). Subsequently, CX43 is translocated from the Golgi apparatus to the cell membrane (Figure 1(C), (c)-(d)) and this dynamic process leads to the formation of discrete gap junctions between adjacent cells, as observed by distinct punctate staining at the cell-cell boundaries between individual AF cells (Figure 1(C), (e)-(f)). This characteristic was not observed for CX26 in AF cells where protein expression was confined to the perinuclear region (Figure 1(C), (g)-(h)).

In order to examine the functionality of gap junctions, we preloaded individual AF cells with two dyes (DiI and calcein) [39, 42] and plated them onto confluent cultures of AF cells. Dye coupling, consistent with the presence of functional gap junctions, was scored by calcein transfer from labeled AF cells to recipient AF cells 4 hrs after-plating. Coupling was observed in an average of 9 ± 1.06  SEM calcein-positive recipient cells coupled to one DiI-positive labeled cell (Figure 1(D), (a–f)).

### 3.2. CX43 Expression in c-kit-Positive and Single-Cell-Derived Clonal AF Cell Populations

**Figure 2:**
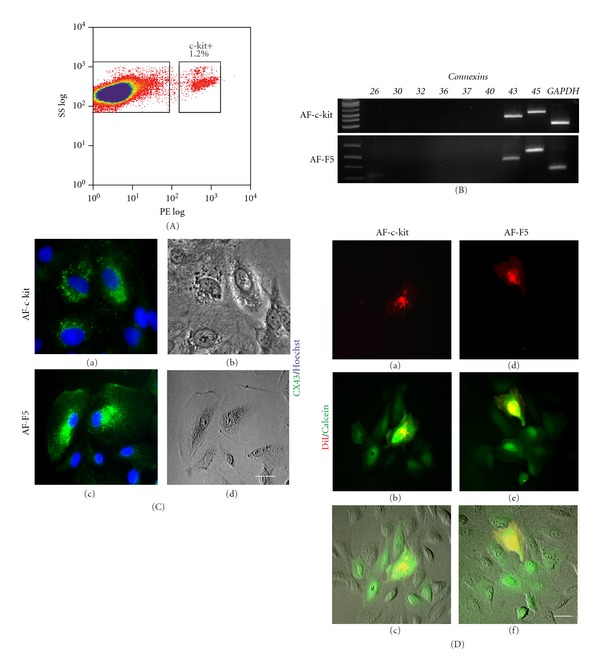
Connexin expression in c-kit-positive and single-cell-derived AF clones. (A) c-kit-positive AF cells were obtained by fluorescence activated cell sorting (FACS). (B) RT-PCR analysis of connexin (*CX*) expression in c-kit-positive (AF-c-kit) and single-cell-derived (AF-F5) AF clones. *GAPDH* transcript was used as an internal control. (C) Immunocytochemistry confirmed the expression of CX43 (green) in AF-c-kit and AF-F5 cultures. Hoechst was used as a counter-stain (blue). (b) and (d) represent the corresponding phase contrast images. Scale bar: 25 *μ*m. (D) AF donor cells were preloaded with DiI (red) and calcein (green) and plated as single cells on cultures of AF cells. Calcein transferred from the donor AF cell to neighbouring cells, confirming the formation of functional gap junctions among AF-c-kit cells as well as AF-F5 cells within 4 hours. Scale bar: 50 *μ*m.

Given the heterogeneity of AF cells [37], we used two established protocols to generate a more homogenous cell population based on single cell cloning [37] and c-kit expression [14], as previously reported. Hence, we generated single-cell-derived clonal AF (AF-F5) and c-kit-positive AF (AF-c-kit, Figure 2(A)) cell populations and found a similar RNA expression profile for *CX43* and *CX45 *(Figure 2(B)) and protein expression for CX43 (Figure 2(C), (a–d)). Dye transfer experiments further confirmed the functionality of gap junctions formed in AF-c-kit (Figure 2(D), (a–c)) and AF-F5 (Figure 2(D), (d–f)) cultures to a similar degree as observed above (data not shown). Hence, AF-F5 cells were used in all subsequent experiments herein.

### 3.3. Intercellular Communication between AF Cells and Cortical Cultures

**Figure 3:**
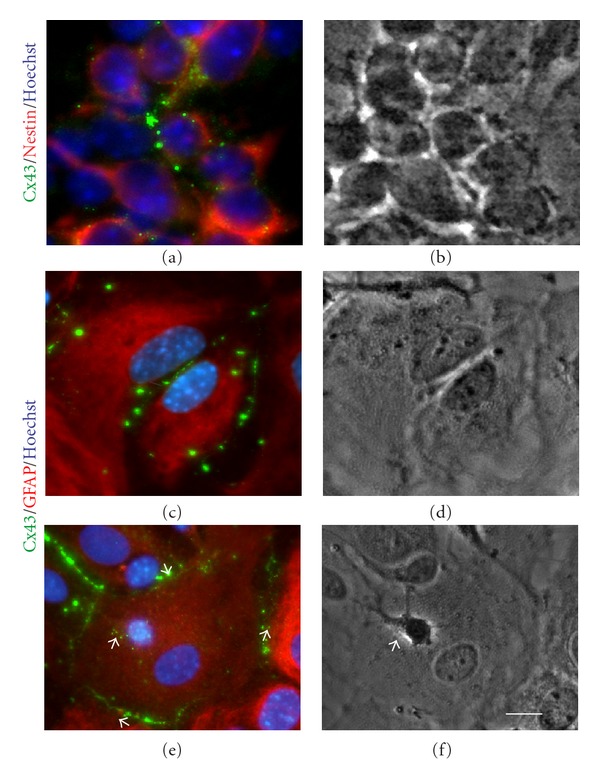
Cx43 expression in mouse cortical cells. (a) Immunocytochemistry showed that Cx43 (green) is expressed in Nestin- (red) positive cortical progenitors. (c) Similarly, abundant levels of Cx43 protein were detected at cell-cell boundaries in cortical astrocytes. (e) Only a limited amount of Cx43 was detected in immature neurons (arrow), whereas astrocytes maintained high degrees of Cx43 expression. (b), (d) and (f) are the corresponding phase contrast images of (a), (c) and (e), respectively. Nuclei were stained with Hoechst (blue). Scale bar: 10 *μ*m.

**Figure 4:**
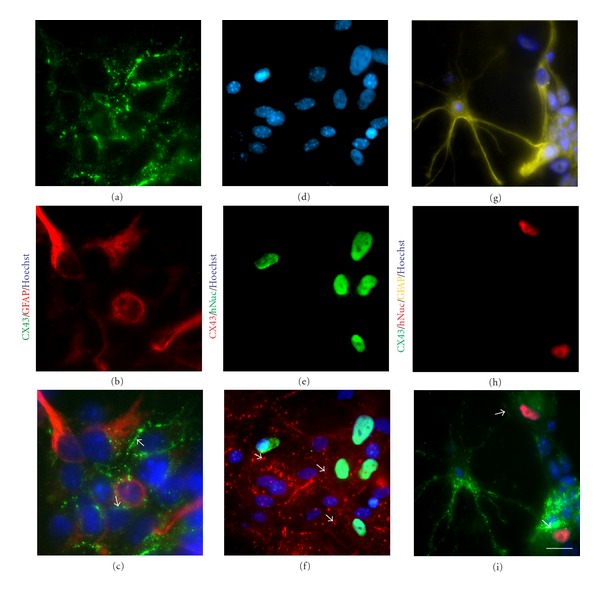
AF cells form gap junctions with cortical astrocytes *in vitro. *Immunocytochemistry showed that AF cells established gap junctions with cortical astrocytes. (a)–(c) CX43 (green) was expressed at the boundary between AF cells and GFAP- (red) positive cortical astrocytes. (d)–(f). In parallel experiments, AF cells labeled with an antibody against human-specific nuclear antigen (hNuc, green) showed discrete, punctuate CX43 (red) staining at the cellular boundary with cortical cells (arrows). (g)–(i). To determine the identity of cortical cells, similar cultures were stained with GFAP (yellow), hNuc (red), and Cx43 (green). Hoechst (blue) was used as a counter-stain. Scale bar: 8 *μ*m (a)–(c), 15 *μ*m (d)–(f), 20 *μ*m (g)–(i).

**Figure 5:**
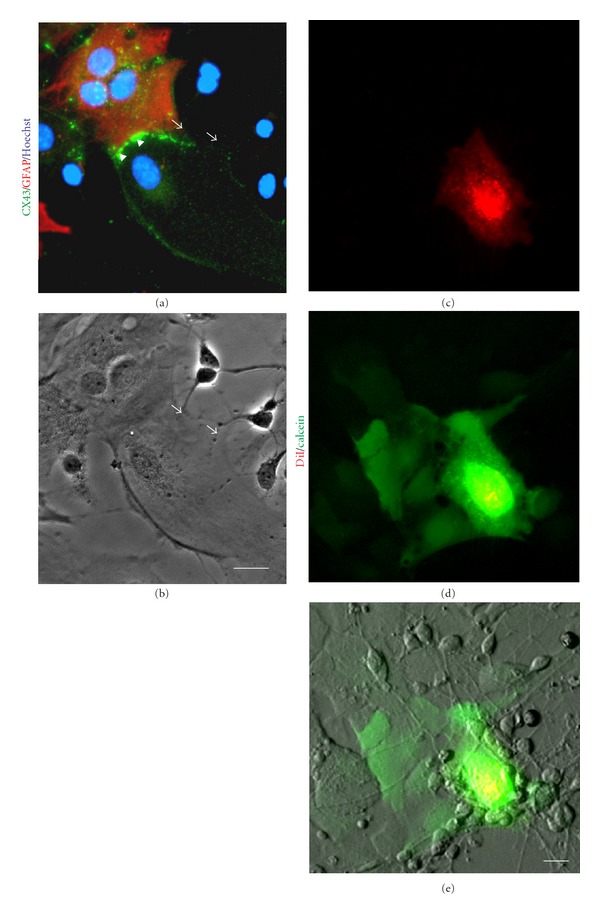
AF cells selectively establish intercellular communication with cortical astrocytes. (a)-(b) Cocultures of AF cells with cortical cells. Immunostaining showed abundant levels of Cx43 protein (green) at the junction between AF cells and cortical astrocytes (GFAP (red); see arrowheads), whereas there was only a limited amount of staining detected at the boundary with neurons (arrows). Scale bar: 10 *μ*m. (c)–(e) AF cells were preloaded with DiI (red) and calcein (green) and plated as a single cell suspension on cortical cultures to examine metabolic coupling. AF cells readily established functional gap junctional communication with astrocytes within 4 hours, as indicated by calcein transfer, a phenomenon not observed between AF cells and neurons. Scale bar: 10 *μ*m.

**Figure 6:**
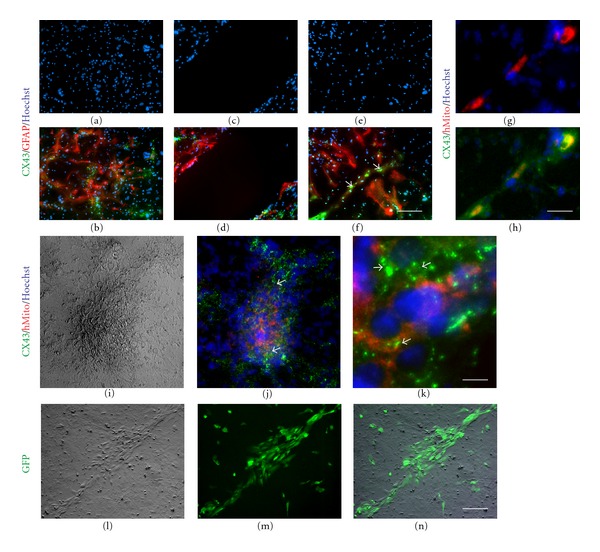
AF cells repair the scratch-induced wound injury in cortical cultures. (a)-(b) A low magnification of confluent cortical cultures stained with Hoechst (a and b, blue), GFAP (b, red) and Cx43 (b, green). (c)-(d) Parallel cortical cultures were subjected to scratch injury and stained with the same markers 24 hrs later. GFAP positive astrocytes were seen at the scratch border (d), without repairing the wound. (e)-(f) In contrast, when AF cells were seeded following scratch, they filled the injury site (f, arrows), maintained CX43 expression (f, green), and facilitated wound repair. (g)–(k) To further identify AF cells located in the injury site, separate cultures were stained with human mitochondrial marker (red), CX43 (green), and Hoechst counter stain (blue). Cx43 was readily expressed at the boundary between AF cells and cortical cells (k). (l)–(n) Live assays, using GFP-tagged AF cells, were also used to confirm wound repair after scratch injury in cortical cultures. Scale bar: 150 *μ*m (a)–(f), 35 *μ*m (g)-(h), 90 *μ*m (I, J), 10 *μ*m (k), 80 *μ*m (l)–(n).

CX43 is considered to be the most ubiquitously expressed member of the connexin family in the mammalian brain and during brain development specifically in neural progenitor cells and astrocytes [46]. Hence, we sought to determine whether AF cells retain CX43 expression and functional gap junctions in cocultures with cortical cells, in particular, with cortical progenitors and astrocytes which are known to express high levels of Cx43 [46] (Figures 3(a)-3(b)) and 3(c)–3(f), resp.). Indeed, when AF cells were seeded on cortical cultures, CX43 was detected at the cell-cell boundary between AF cells and GFAP-positive cortical astrocytes (Figures 4(a)–4(c)), arrows). In parallel cocultures, AF cells were distinguished from mouse cortical cells, using a human specific nuclear antigen (hNuc) (Figures 4(d)–4(i)) or in some instances with a human-specific mitochondrial antigen (see Figure 6). Quadruple staining confirmed that the cortical cells, which formed gap junctions with AF cells, were GFAP-positive astrocytes (Figures 4(g)–4(i)). Interestingly, when AF cells were cultured alone, CX43 expression was predominantly cytoplasmic and perinuclear (Figure 1(C), (a–d)); however, AF cells cocultured with cortical cultures showed more membrane-bound CX43 staining between adjacent cortical astrocytes (Figure 4(c), 4(f), and 4(i), arrows). Even in the presence of cortical neurons, the majority of CX43 expression was observed at the boundary between AF cells and GFAP-positive astrocytes (Figures 5(a)-5(b)), arrowheads); whereas a negligible amount of Cx43 protein was observed between AF cells and neurons at the interface of neurites and the AF cell membrane (Figures 5(a)-5(b)), arrows). Although the staining results suggest that AF cells retain CX43 expression to mediate gap junction formation with target cells, we performed dye transfer experiments (as outlined earlier) to confirm functional gap junction formation and intercellular connections between AF cells and cortical cultures. Coupling was observed in an average  40 ± 15.21  SEM calcein-positive recipient cells coupled to a single calcein-positive, DiI-positive donor AF cell after 4 hours (Figures 5(c)–5(e)). Of note, in earlier cortical cultures (2 days *in vitro*), AF cells were able to establish functional communication with immature cortical neurons expressing much lower levels of CX43 (data not shown); however, functional communication was largely observed between AF cells and cortical astrocytes in later cultures (Figures 5(c)–5(e)). In support of this observation, Cx43 protein expression has been known to decrease significantly following neuronal differentiation [39, 47].

### 3.4. Cx43 Expression during Injury

As a response to brain injury, astrocytes proliferate and infiltrate the damaged region in an effort to preserve neural tissue and restrict inflammation [48]. Since Cx43 is the main protein expressed in both astrocytes and AF cells and results in functional gap junctional intercellular communication, we examined the interaction of AF cells with host cells in both *in vitro* and *in vivo* brain injury models. More specifically, we used an *in vitro* scratch wound model using cortical cultures, a well-characterized model to investigate the astrocytic response to mechanical injury [49], as well as in an *in vivo* surgically induced brain injury model targeting the primary motor cortex [50].

Cortical astrocytes expressed abundant levels of Cx43 (Figures 6(a) and 6(b)). Although astrocytes maintained Cx43 expression following a scratch-induced injury, they did not demonstrate the capacity to repair the wound within the first 24 hrs (Figures 6(c) and 6(d)). In fact, glial processes have been shown to protrude into the injured region with astrocytes filling the gap approximately 72 hrs after-injury [51]. In contrast, AF cells seeded onto injured cortical cultures 15 minutes after scratch were able to adhere to the denuded area and facilitate the repair within 24 hrs (Figures 6(e)-6(f)), arrows). In order to confirm that seeded AF cells were able to reestablish connectivity with cortical astrocytes, via Cx43-mediated gap junction formation, we labeled AF cells with a human mitochondrial antibody (hMito) and examined CX43 expression between AF and cortical cells (Figures 6(g)–6(k)). CX43 was readily expressed at the boundary between AF cells and cortical astrocytes (Figure 6(k)). Complementary to these experiments, we performed a live assay by seeding GFP-tagged AF cells following injury and confirmed that AF cells readily filled the injury site, resulting in wound closure (Figures 6(l)–6(n)).

**Figure 7:**
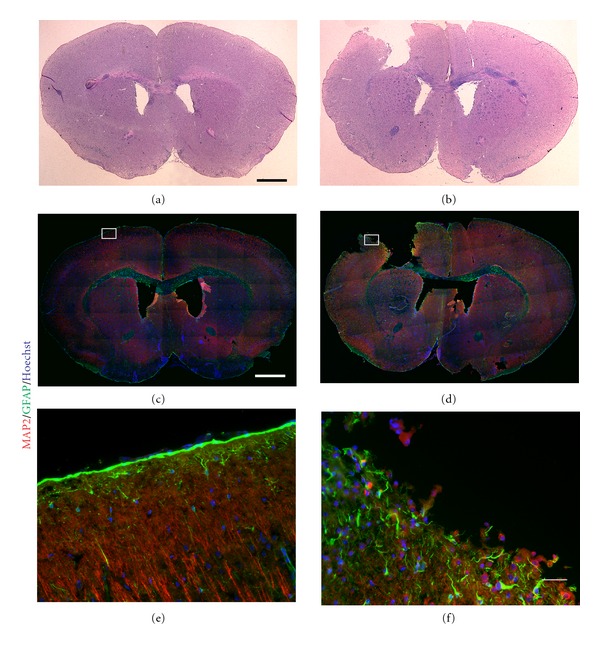
Cellular architecture in normal and injured motor cortices. (a)-(b) Hematoxylin and eosin (H&E) staining of coronal sections from mice subjected to sham surgery (a) and motor cortex injury (b). (c)-(d) Mosaic confocal images of adjacent sections show MAP2- (red) positive neurons and GFAP (green) astrocytes in sham (c) and injured (d) brains. (e)-(f) Higher magnifications of the insets in (c) and (d). In contrast to the well-organized architecture in the control motor cortex (e), there was a significant loss in the number of neurons, associated with an increase in astrogliosis in the injured cortex. Hoechst (blue) was used to label the nuclei. Scale bars: 1000 *μ*m (a)–(d), 100 *μ*m (e)-(f).

**Figure 8:**
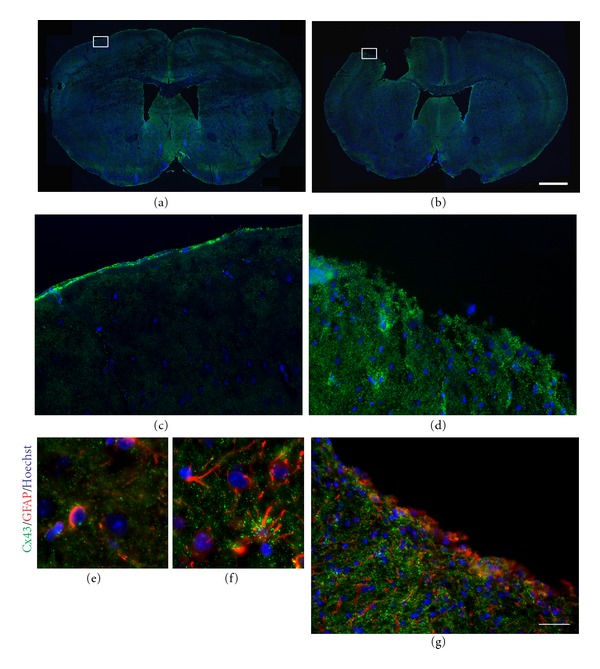
Cx43 expression in the control and injured motor cortices. Immunohistochemistry of Cx43 (green) in the brains of mice subjected to sham surgery (a,c,e) and motor cortex lesion (b, d, f, g). (a)-(b) The stitched confocal images show the expression of Cx43 in the control (a) and injured (b) brains. (c)-(d). Higher magnification images of the areas of motor cortex indicated by insets in (a) and (b) are shown in (c) (sham) and d (lesion). Increased Cx43 staining was observed in the cortex adjacent to the injury site compared to sham cortex. (e)–(g). Double-labeling with GFAP (red) indicated that Cx43 (green) was expressed mainly in astrocytes (red), and more abundant in the injured cortex (f, g), compared to sham cortex (e). Nuclei were stained with Hoechst (blue). All the sections were coronal. Scale bar: 1000 *μ*m (a)-(b), 100 *μ*m (c, d, g); 10 *μ*m (e, f).

Using a mouse model of brain injury, the motor cortex was injured, as previously described [44, 50], resulting in a cavity that forms as a result of tissue loss and cell death that ensued after the injury (Figures 7(b), 7(d), and 7(f)) compared to sham (uninjured) brains (Figures 7(a), 7(c), and 7(e)). Compared to the organized architecture of neurons and astrocytes in the control brain (Figure 7(e)), the damaged cortex showed significant neuronal loss (approximately 210,000 neurons from a total of 350,000 cells), a disarray of neurite extensions, and a significant infiltration of astrocytes to the injured area (Figure 7(f)). Since astrocytes exhibit a high degree of coupling through gap junctions, composed mainly of Cx43 [52], we examined the immunohistochemical distribution of Cx43 in the injured brain. Indeed, compared to the uninjured brain (Figures 8(a) and 8(c)), abundant levels of Cx43 were detected at the perimeter of the injury site (Figures 8(b) and 8(d)). Semiquantitative immunohistochemistry confirmed increased Cx43 (40%) and GFAP (65%) expression in the damaged motor cortex (Figures 8(f)-8(g)), compared to the corresponding region in the sham brain (Figure 8(E)). Areas of intense Cx43 puncta were specifically observed in astrocytes within close proximity to the injury site (Figures 8(f)-8(g)). The upregulation of Cx43 enhances intercellular communication in the brain and may facilitate the delivery of beneficial factors to the injured brain.

**Figure 9:**
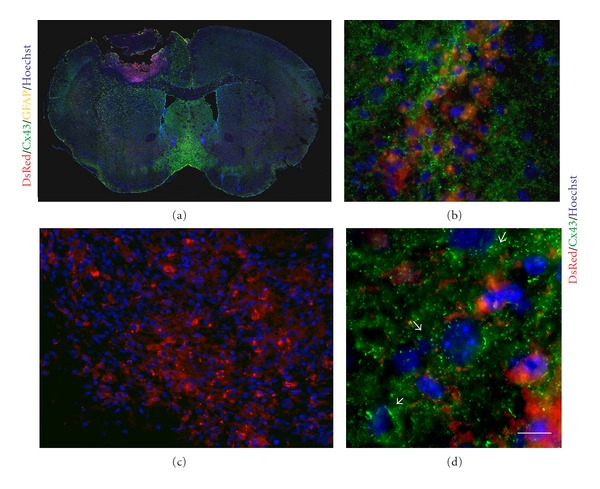
Gap junctions between AF cells and astrocytes following cell implantation into the injured motor cortex. (a) Stitched confocal image of mouse brain after motor cortex injury and receiving AF-DsRed (red) cell implant. Immunohistochemistry shows the distribution of Cx43 protein (green) at the interface between the graft and injured host tissue. (b) A higher magnification of the implant confirmed that AF-DsRed cells could be easily traced in the injured brain. (c)-(d) Intense Cx43 expression (green) was observed at the site of implantation. More specifically, distinct Cx43 immunostaining was detected at the junction between AF-DsRed (red) and cortical cells (see arrows). Hoechst was used as a counter stain (blue). All the sections were coronal. Scale bars: 1000 *μ*m (a), 100 *μ*m (b), 50 *μ*m (c), 7 *μ*m (d).

To determine whether gap junctions form between AF and cortical cells *in vivo* and hence hold translational relevance, we implanted AF cells labeled with DsRed (AF-DsRed) into the injured motor cortex (Figures 9(a)-9(b)). Immunohistological analysis showed abundant Cx43 expression in the implanted area, as determined by DsRed and CX43 (Figures 9(c)-9(d)), whereas no AF-DsRed cells were found on the contralateral side which did not receive an injury/implantation of cells (Figure 9(a)). At 12 days after implantation, the majority of AF-DsRed cells were located within the injury injection site and needle tracks (Figure 9(c)), accompanied by CX43 expression surrounding the injection site. In fact, Cx43 expression was observed at the junction of AF-DsRed and neighbouring cells, as seen at higher magnification (Figure 9(d)). Although the long-term outcome of AF cell implantation into the motor cortex injury model has not been examined, abundant Cx43 expression between cortical astrocytes and AF cells suggests intercellular communication and potentially reconstruction of neural circuitry after AF cell engraftment.

## 4. Discussion

The development of functional grafts in the CNS is limited by the potential absence of intercellular communication between grafted donor cells and host tissue. Thus, it is expected that enhancement of connexin-mediated intercellular gap junction formation would result in improved cell-cell communication between host and graft cells and increase transplantation success rate. Since AF cells have attracted a great deal of attention as an alternative source of donor cells for cell-based therapies, we examined their potential to form gap junctions and found that AF cells express abundant levels of CX43. Using well-established methods to isolate homogenous c-kit and single-cell-derived AF cell clones, we demonstrated that CX43 may play an important role in intercellular communication among these cells. These results are in agreement with the expression of CX43 in ES cells [53] and other cell lines with stem cell characteristics such as NT2/D1 [42] and P19 [54] cells. This is not surprising, as results from several laboratories have established that CX43 is the most prevalent connexin protein in vertebrates (see [55] for review). Cx43 is expressed in at least 34 tissues and 46 cell types [56, 57], and it plays a critical role in coordinating tissue functions and cellular homeostasis.

In the brain, Cx43 is highly expressed in the developing cortex and maintains its expression in cortical astrocytes throughout adulthood [55, 58, 59]. The presence of active gap junctions in astrocytes allows the regulation of glucose and oxygen delivery to neurons for their energetic and metabolic needs [60, 61]. For instance, Cx43 mediates the transfer of lactate from astrocytes to neurons as an energy substrate and facilitates the synthesis of neurotransmitters for synaptic activity [61, 62]. Similarly, the delivery of glucose and oxygen from the blood to the brain is regulated through an astrocytic network, which is dependent on Cx43, as demonstrated by knock-out experiments [61]. Hence, the ubiquitous expression of CX43 in AF cells also makes these cells suitable to serve as a platform to deliver beneficial factors through direct communication with brain cells. AF cells can potentially help modulate inflammatory cues and buffer pathological stimuli in the brain following injury as well as other neurological diseases. The rapid subcellular translocation of CX43 from the perinuclear compartment to the membrane boundary between AF cells and astrocytes may enhance the reestablishment of a homeostatic state in the brain after injury. Interestingly, CX43 has also been observed at the borders of AF and cardiac cells, following transplantation into the heart [63], further supporting the application of AF cells in regenerative medicine through the formation of functional gap junctions.

Changes in both spatial and temporal CX43 protein expression are seen following various types of CNS pathologies such as ischemia, neurodegenerative disorders, and traumatic injury [64]. In brain injury an infiltration of Cx43-positive reactive astrocytes is readily observed in the injured core [65]. The induction of CX43 expression is a substantial factor in the astroglial response and potentiates intercellular signal transduction via gap junctions following injury [33, 65]. Consistent with these observations, we found Cx43 expression in astrocytes at the site of injury. Following injury, the increased expression of CX43 in astrocytes and hence the number of gap junction plaques formed at the interface between neighbouring cells may facilitate the formation of gap junctions with graft cells implanted in close proximity to the injury site. Hence, by introducing AF cells, the expression of CX43 between graft and host cells enables formation of gap-junctions which would aid in establishing communication between AF and CNS cells for delivery of beneficial factors and drugs. GJIC between grafted neural stem (NS) cells and brain cells [33] appears to be an essential participant in the neuroprotective effect associated with NS cell engraftment, particularly at the connexin-associated gap junction interface. Utilizing NS cells grafted into an *ex vivo* model system for striatal tissue, Jäderstad et al. [66] found that CX43 expression transiently peaked in host cells following traumatic stimulation, suggesting a window of opportunity for NS cells to establish gap junctions with the host tissue and rescue the damaged cells. Since AF cells express high levels of CX43 and form functional gap junctions, they have the capacity to mimic a similar connexin-mediated rescue during this critical time frame. In fact, preventing damaged cells from dying has emerged as one of the possible benefits of cell transplantation [1, 2, 25]. Furthermore, cell-cell coupling has been regarded as an early form of communication that precedes and acts as a template to establish electrochemical synapses later on [33]. In this instance, implanted AF cells expressing CX43 may form gap-junctional coupling with astrocytes to possibly preserve neurons at the injury site. In support of this view, the role of astrocytes in early stages of neuroprotection is gaining more recognition, and initial AF-astrocyte interactions may play an important role during early stages in graft-host interactions [33]. This notion is further substantiated by findings *in vitro,* which confirm that astrocytic Cx43 gap junctions and hemichannels may remain functionally open following injury and *in vivo* work, which has shown significant changes in both spatial and temporal Cx43 expression following various models of CNS injury (reviewed in [67]).

It remains to be elucidated how astrocytic gap junctions contribute to neuroprotection in the context of regenerative medicine. Hence, further investigation of intercellular communication between AF and host cells may facilitate the use of these cells for therapeutic purposes.

## Figures and Tables

**Table 1:** Sequence and annealing temperatures for RT-PCR.

Designation	Sequence (5′-3′)	Annealing temp. (°C)	Amplicon size (bp)	Ref.
*CX26-F*	CTGCAGCTGATCTTCGTGTC	55	308	[18]
*CX26-R*	AAGCAGTCCACAGTGTTG			
*CX30-F*	GCTACCTGCTGCTGAAAGTG	58	326	[40]
*CX30-R*	CGTTGTGTATGAATGGAGCA			
*CX32-F*	GACAGGTTTGTACACCTTGC	58	500	[41]
*CX32-R*	CGTCGCACTTGACCAGCCGC			
*CX36-F*	AACGCCGCTACTCTACAGTCTTCC	55	268	[20]
*CX36-R*	GATGCCTTCCTGCCTTCTGAGCTT			
*CX37-F*	GTTGCTGGACCAGGTCCAGG	58	416	[40]
*CX37-R*	GGATGCGCAGGCGACCATCT			
*CX40-F*	GTACACAAGCACTCGACCGT	58	509	[40]
*CX40-R*	GCAGGGTGGTCAGGAAGATT			
*CX43-F*	CAATCACTTGGCGTGACTTC	58	408	[40]
*CX43-R*	GTTTGGGCAACCTTGAGTTC			
*CX45-F*	GGAGCTTCCTGACTCGCCTGC	58	467	[40]
*CX45-R*	CGGCCATCATGCTTAGGTTT			
*GAPDH-F*	CATGACCACAGTCCATGCCATCACT	58	461	
*GAPDH-R*	TGAGGTCCACCACCCTGTTGCTGTA			
